# Utilization of quarry dust and periwinkle shell ash in concrete production

**DOI:** 10.1007/s11356-024-34990-4

**Published:** 2024-10-02

**Authors:** Chioma Emmanuella Njoku, Anthony Chibuzo Ekeleme, Benjamin Nnamdi Ekwueme, Chukwudike Onuoha, Ebube Prince Onuzulike, Wisdom Chibundu, Kooffreh Okon, Chibuike Christopher Ozoh

**Affiliations:** 1 Department of Materials and Metallurgical Engineering, Federal University of Technology Owerri, P.M.B 1526, Owerri, Imo State Nigeria; 2 Department of Civil Engineering, Abia State University, P.M.B 2000, Uturu, Abia State Nigeria; 3 Department of Civil Engineering, Gregory University, P.M.B 1012, Uturu, Abia State Nigeria

**Keywords:** Concrete, Periwinkle shell, Quarry dust, Partial replacements, Compressive strength, Supplementary cementitious materials

## Abstract

The usage of plentiful raw discarded resources in the manufacturing of concrete has proven to be a sustainable and environmentally beneficial method of making concrete for a variety of purposes. In this study, the physical and mechanical properties of concrete made by partially and fully substituting fine aggregates and ordinary Portland cement with periwinkle shell ash and quarry dust (5%, 10%, 15%, 20%, and 100%), respectively, were examined. The ratio of water to cement utilized for the concrete mixture, 1:2:4, was 0.60. Fresh concrete underwent a slump test, and then 150-mm cubes of cured concrete were subjected to density, compressive strength tests, and morphological and structural property characterizations. The concrete without the waste materials gave an optimum compressive strength of 22.9 N/mm^2^ as opposed to those that were partially replaced, having 18.8–15.1 N/mm^2^. The concrete samples with full replacements of periwinkle shell ash and quarry dust have compressive strengths lower than 13.8 N/mm^2^. All the concrete samples produced with partial and full replacements are in the class of normal concrete, but only those with partial replacements of up to 20% can be utilized for load-bearing and non-load-bearing applications. Opting for these alternative waste materials implies taking steps towards creating a cleaner and healthier planet for now and the future.

## Introduction

Concrete is used and applied in various ways in the building sector. It is the most common and adaptable material used in many different types of residential and commercial construction works worldwide. This is because of its inherent properties, durability, and increased strength with aging, high reflectivity, and ability to stand against natural disasters. Cement, water, and aggregates are the primary ingredients used in concrete making (Raheem et al. 2021). Sadly, the primary components utilized in the manufacture of conventional concrete are harmful to the environment. The cement industry is faced with the challenge of the reduction of CO_2_ emissions and also the need to meet high global demands. The production of ordinary Portland cement involves significant energy consumption and noisy activities, such as operating electric engines, crushers, blowers, and milling machines (Ruslan et al. 2021). Additionally, this process releases a high percentage of CO2 into the atmosphere, up to 8% of global carbon dioxide emissions, and about 90% of this comes from the production of clinker, the primary ingredient that provides strength to concrete (K. Ogundipe et al. 2020). About 4.1 billion tons of cement were estimated to have been produced globally in the year 2022, and each ton of cement produced releases 0.6 t CO2 of each ton produced which largely contributes to the issue of global warming (M. Garside 2023; Soomro et al. 2023). In a bid to achieve net zero emissions by the year 2050, measures are put in place to reach the desired goal by utilizing several supplementary cementitious materials to partially or fully replace cement in concrete production. Attempts are made in this study to address Goal 11.6.1 of the Sustainable Development Goals, which aims to manage waste by collecting and properly disposing of solid waste generated by cities by 2030. It also supports the objective of ensuring access to secure, affordable housing and essential services for everyone and also improving informal settlements by 2030, as outlined in Goal 11.1 (UNGA 2020).

Supplementary cementitious materials (SCMs) include materials that add to the properties of cement-based materials hydraulically or by pozzolanic action. The supplementary cementitious materials can consist of the following materials; palm kernel ash, rice husk and seashell ash, ground blast furnace slag, and silica fume (Eziefula et al. 2018, 2020). The use of these SCMs brings about cost reduction, improvement in the technical features of the concrete, and more especially in the lessening of pollution and management of waste. Seashells have always been processed into the ash and used as supplementary cementitious materials for they are largely abundant. The quantity of seashells deposited as waste is so much because the focus has been on the harvesting of the snail meat without due consideration to the disposed shells. It has been reported that about eight million tons of waste seashells (shrimps, crabs, and lobster shells) and also more than ten million tonnes of waste mollusk seashells are produced every year worldwide (Topić Popović et al. 2023). Periwinkle is a seafood and belongs to the gastropod mollusks. They are widely available in the rivers and coasts in Nigeria, and when eaten, their shells which are majorly composed of CaCO_3_ (up to 96%) are discarded as waste (Luhar et al. 2019). There are very scanty reports on the amounts of periwinkle shells in Nigeria. However, Ekop et al. (2021) reported that up to 40.3 tons of periwinkle are harvested yearly from about 35 coastal areas in the Nigerian states of Delta and Rivers. This amount excludes the other states in Nigeria, which suggests that the quantity of waste generated from the periwinkle shell food nationally is enormous. The shells are frequently disposed of in large amounts in open spaces and landfills, which causes serious pollution problems. To convert the discarded periwinkle shell waste into a useful resource, researchers have been working on its use, particularly in concrete production. The periwinkle shells/ash has been studied to envisage its potential usage in concrete production as a partial replacement for fine aggregates, coarse aggregates, and cement. In a study (Prusty and Patro 2015), the properties of fresh and hardened concrete containing agro-wastes as partial replacements of coarse aggregate were reviewed. The study concluded that periwinkle shells can be used to produce lightweight aggregates. It was also found that with approximately 75 percent replacements, the concrete can achieve compressive strengths of more than 20 MPa. Bamigboye et al. (2016) employed periwinkle shell as a fractional substitute for both fine and coarse aggregates, reporting that up to 30 wt% of the periwinkle shell for the fine aggregate as well as the coarse aggregates will result in compressive strengths of up to 20 N/mm^2^. They reported that as the periwinkle shells increased from 1 to 100%, it resulted in a decrease in slump values, and that up to 6.8% was saved in cost when 30-weight percent periwinkle shells were utilized in concrete making. In the investigation carried out by Afolayan et al. (2019) on the replacement of cement partially with periwinkle shell ash, their results showed an increase in compressive strength for 5 wt% periwinkle shell ash and 40 mm sisal length, after which the compressive strength decreased on the sisal fiber-reinforced concrete. The authors found that as the curing time increased to 28 days, the tested samples showed the highest compressive strengths. A compressive strength of up to 28.8 N/mm^2^ was achieved with the combination of sisal fiber and periwinkle shell ash. Umoh and Ujene (2015) studied the effect of sodium nitrate on the compressive and tensile splitting strengths of cement concrete blended with periwinkle shell ash. The authors resolved that adding 2% NaNO_3_ and 30 wt% periwinkle shell ash resulted in optimal compressive and tensile splitting strengths of the produced concrete.

Sand mining for construction has become a quiet environmental challenge worldwide that goes on unmonitored by the government. It has been reported that over 50 billion tons of sand is mined yearly worldwide, and the excavation has caused much havoc on the supply of food and water, the lives of aquatic animals, and the entire human well-being (Luhar et al. 2019; Sennott 2023). To produce eco-friendly and sustainable concrete, the waste materials that cause environmental pollution have the possibility of being used as partial substitutes for sand in concrete production and also reduce sand excavation issues. Some of these waste materials include sea shells, agricultural wastes, fly ash, quarry dust, waste plastics, steel slag, sawdust, and rice husk ash amongst others (Meko and Ighalo 2021). Quarry dust is released as a result of crushing operations, and it poses serious pollution to water, land, and air. In a bid to put them to good use, they are utilized in replacing fine aggregates in concrete production. Scholars have reported enhancement in the mechanical properties of concrete that replaced fine aggregates with quarry dust (Febin et al. 2019; Oorkalan et al. 2020; Ponnada et al. 2020).

The production of concrete can be made more environmentally friendly by utilizing multiple waste materials while also reducing costs. Most studies have shown that combining two or more waste materials as aggregates in producing sustainable concrete for buildings can enhance certain properties under investigation. Martínez-García et al. (2024) studied the limit of replacing mussel shell aggregates in mortars. They reported that using 25% of mussel shells as replacements for conventional aggregates would yield coating mortars that can fulfill the standard requirements for various applications. They also highlighted that the replacement with mussel shells will bring about a great reduction in environmental pollution. Ogundipe et al. (2021) investigated the strength characteristics of lightweight concrete made by partially replacing granite with palm kernel shells and periwinkle shells. The authors found that by combining periwinkle shells and palm kernel ash in a 5 wt% ratio each, they could create lightweight concrete that can still support reasonable loads. There is no existing literature on the use of quarry dust and periwinkle shell ash as individual constituent replacements in the same sustainable concrete. There is also a need to add to the existing knowledge and create enough data on the usage of these discarded/waste materials for making of concrete because of how important concrete structures are to humans and the increasing population growth that places so much demand on their diverse applications. Thus, the study is aimed at examining the usage of quarry dust (QD) and periwinkle shell ash (PSA) as partial or full replacements for fine aggregate and cement, respectively.

## Materials and methods

### Materials

**Fig. 1:**
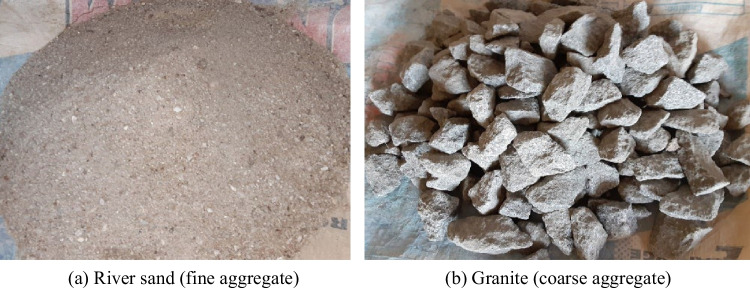
Fine and coarse aggregates. **a** River sand (fine aggregate). **b** Granite (coarse aggregate)

**Fig. 2:**
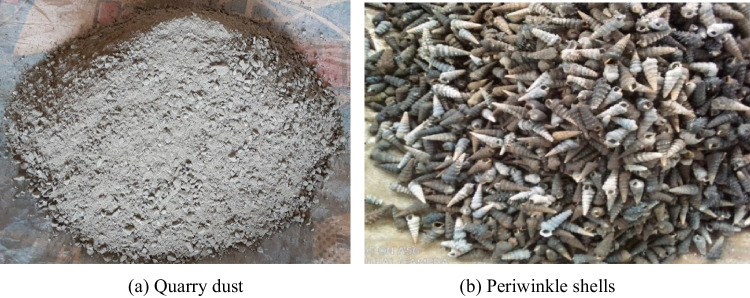
Waste materials added. **a** Quarry dust. **b** Periwinkle shells

The basic materials complied with outlined standards (BS 2002). Dangote ordinary Portland cement (OPC), grade 42.5R, with initial and ultimate settling times of 75 and 150 min, respectively, and a consistency of 28%, was the binder that was utilized. The materials utilized were granite, river sand, and periwinkle shells from Owerri, Imo State, and quarry dust from Isiagu, Ebonyi State. The periwinkle shells were washed, sun-dried, calcined in a muffle furnace at 1000 °C for 1 h, and then ground to a particle size of 4.5 mm with a grinding machine at Eziobodo market in Owerri. Portable water was obtained from the Federal University of Technology Owerri and met the standard (BS 2002). The samples of the granite, river sand, quarry dust, and periwinkle shells are shown in Figs. 1 and 2.

The compositional analysis of the periwinkle shell ash was obtained using X-ray fluorescence (XRF) analysis. The periwinkle shell ash underwent a process where it was first crushed with an electric crusher and then pulverized using a Herzog Gyro-mill (Simatic C7-621). The pulverized samples were then made into pellets by grinding 20 g of the sample with 0.4 g of stearic acid for 1 min. A gram of stearic acid, which acts as the binding agent, was weighed into an aluminum cup, and the cup was later filled with the sample to a specific level. The cup was then taken to the Herzog pelletizing equipment and subjected to a pressure of 200 KN for 60 s. The resulting pelletized sample, which was 2 mm in size, was placed in a sample holder of the X-ray fluorescent (Phillips Pw-1800) for analysis. The chemical compositions of ordinary Portland cement, periwinkle shell ash, and quarry dust used in concrete production are revealed in Table 1. Whereas the physical characteristics of the constituents are presented in Table 2, the sieve analysis of the aggregates was in line with and according to the limits specified by ASTM C90 (2017). The periwinkle shell ash has the same major constituents as cement, including CaO, SiO_2_, Fe_2_O_3_, and Al_2_O_3_, as shown in Table 1. The periwinkle shell ash has high contents of CaO and SiO_2_, similar to that of cement, which makes it a pozzolan (David Offiong and Edem Akpan 2017; Eziefula et al. 2020). However, it may not be as reactive as cement but can be a supplementary cementitious material. This suggests that periwinkle shell ash can partially replace cement in concrete production.

**Table 1:** Chemical compositions of the constituent materials used

Constituents	Oxides and percentages										
	Al_2_O_3_	CaO	Fe_2_O_3_	K_2_O	SiO_2_	MgO	Na_2_O	SO_3_	MnO_3_	TiO_2_	LOI
Periwinkle shell ash	2.60	66.98	3.40	0.30	20.50	0.05	0.002	1.00	0.22	0.170.78	
^a^Cement	3.91	66.55	3.62	0.39	19.21	1.17	0.40	3.23	-	-	-
^b^Quarry dust	15.52	14.30	6.53	4.35	45.4	3.54	-	0.55	-	2.65	-

(^b^Kufre Etim et al. 2021; ^a^Sathiparan and De Zoysa 2018)

**Table 2:** Physical properties of the constituents

Properties	Constituents				
	OPC	Periwinkle shell ash	Quarry dust	River sand	Granite
Specific gravity	3.13	2.00	3.21	2.60	2.60
Bulk density (kg/m^3^)	830	727	2147	1397	2711
Particle size (mm)	5.00	4.50	7.50	6.80	20.00
Fineness modulus (%)	2.5	2.62	2.82	2.50	6.78
Color	Greenish-grey	White-ash	Grey	Pale green	Gray/white/black

### Method

A scanning electron microscope (model; JEOL JSM-7600F) was utilized in studying the morphological characteristics of the periwinkle shell ash and quarry dust. Also, the spectra of the bonds in the PSA and QD were obtained with a Fourier Transform Infrared (FTIR) Spectroscopy (Agilent Cary 630 model) with an attached diamond attenuated total reflectance, having scanning ranges from 4000 to 400 cm^−1^, 32 scans, and resolution of 8 cm^−1^.

The concrete laboratory of the Department of Civil Engineering in the Federal University of Technology Owerri, Nigeria, was where the materials were mixed, batched, and tested following standards. The mix designations and batching of the material elements are presented in Tables 3 and 4, respectively, and a mix ratio of 1 to 2 is to 4 (cement/PSA to sand/QD to granite) and 0.60 water to cement ratio was employed. The concrete was cast in cubes using metallic molds of 150 mm and then cured for 7, 14, 21, and 28 days.

**Table 3:** Concrete mix designations and constituent percentages

Concrete mix designation	Cement (1) (%)	PSA (%)	Sand (2) (%)	QD (%)	Granite (4) (%)	Water:cement
A (control)	100	0	100	0	100	0.6
B	95	5	95	5	95	0.6
C	90	10	90	10	90	0.6
D	85	15	85	15	85	0.6
E	80	20	80	20	80	0.6
F	75	25	75	25	75	0.6
G	70	30	70	30	70	0.6
H	65	35	65	35	65	0.6

**Table 4:** Amounts of concrete constituents used for 1 m^3^ of concrete

Mix designation	Cement (kg/m^3^)	PSA (kg/m^3^)	Sand (kg/m^3^)	QD (kg/m^3^)	Granite (kg/m^3^)	Water (kg/m^3^)	Water:cement
A	118.6070	0	399.12	0	1549.07	71.2	0.6
B	112.6763	5.9304	379.164	19.956	1549.07	71.2	0.6
C	106.7463	11.8607	359.164	39.912	1549.07	71.2	0.6
D	100.8159	17.7911	339.252	59.868	1549.07	71.2	0.6
E	94.8856	23.7214	319.296	79.824	1549.07	71.2	0.6
F	88.9552	29.6518	299.340	99.78	1549.07	71.2	0.6
G	83.0249	35.5821	279.384	119.736	1549.07	71.2	0.6
H	77.0945	41.5125	259.428	139.692	1549.07	71.2	0.6

The slump test was done on the fresh concrete in line with ASTM C-143 standard (ASTM C143-17 2017), the test on the compressive strength of the cured concrete was performed according to the standard specified by BS 12390 (BS 2009), and the density was done in line with the specified ASTM standard (ASTM 2006). Results were presented as average values after triplicate runs.

## Results and discussion

### Scanning *electron* microscopy

**Fig. 3:**
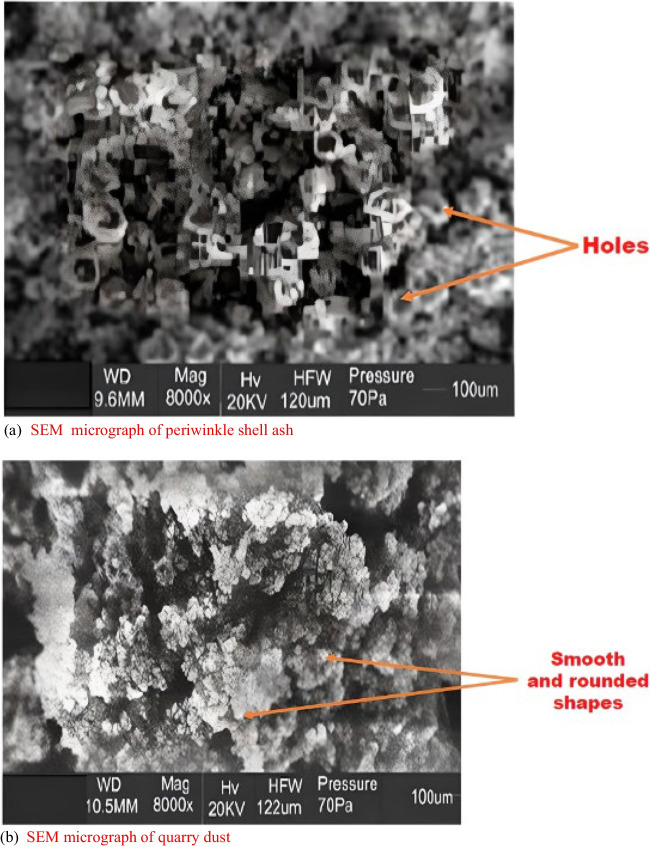
SEM micrographs of the added waste materials **a** periwinkle shell ash and **b** quarry dust

Figure 3a and b reveals the periwinkle shell ash and the quarry dust micrographs, respectively. The scanning electron microscope (SEM) micrographs showed that the PSA displays irregular and porous shapes with varying pore sizes and distribution throughout the matrix. This inherent porosity is advantageous as it facilitates improved bonding between the PSA and the cement matrix at a microscale level. Ultimately, this enhanced bonding mechanism contributes to increased mechanical interlocking, thereby enhancing the strength of the concrete material. This ability to interlock with other constituents is the function of ordinary Portland cement. Then, the quarry dust (Fig. 3b) has smooth and spherical particles, enhancing the workability of the concrete. The scanning electron microscope (SEM) also reveals a dense morphology with reduced porosity. This decrease in porosity would result in reduced water absorption and increased durability of the concrete, making it more resilient to environmental effects (Alizada et al. 2023).

### Fourier transform infrared (FTIR) spectroscopy

**Fig. 4:**
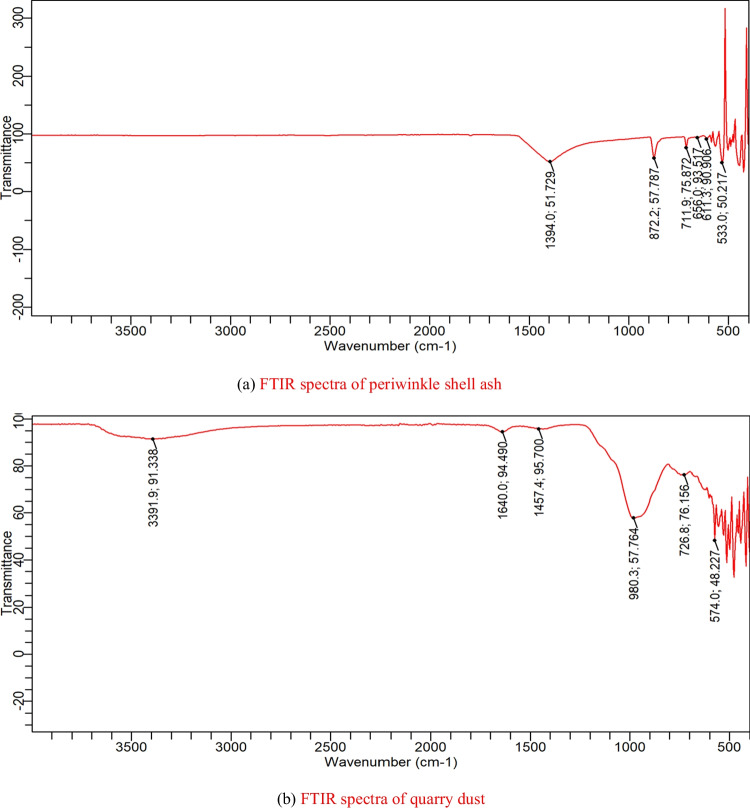
**a** FTIR spectra of periwinkle shell ash. **b** FTIR spectra of quarry dust

The periwinkle shell ash FTIR spectra are revealed in Fig. 4a, while that of the quarry dust are in Fig. 4b, and the recorded range is from 4000 to 400 cm^−1^.

The observed peaks on the periwinkle shell ash spectrum and their equivalent band groups include peak 1394 cm^−1^, linked to C- H bending groups as the PSA is a highly carbon-based material. Peaks 872 cm^−1^ and 711 cm^−1^ match the C–O vibrations of carbonates. Peaks 656 cm^−1^ and 611 cm^−1^ correspond to the S–O stretching of the sulfates, and peak 533 cm^−1^ for Si–O stretching (Attah et al. 2022; Tararushkin et al. 2020). The minerals associated with the respective wavelengths and functional groups of the periwinkle shell ash are presented in Table 5. The periwinkle shell ash has functional groups and minerals comparable to those in Portland cement, especially the tricalcium silicate. Therefore, the PSA can be a suitable substitute for Portland cement in concrete production (Singh et al. 2021).

**Table 5:** Minerals and associated band groups in PSA

Minerals	Wave number (cm^−1^)	Band group
Carbon-based	1394	CH and CH_2_ bendings
Vaterite	872	C–O vibrations
Vaterite	711	C–O vibrations
Bassanite	656	S–O stretching
Anhydrite	611	S–O stretching
Alite (tricalcium silicate)	533	Si–O stretching

(Fernández-Carrasco et al. 2012; Tararushkin et al. 2020).

The observed peaks on the quarry dust and their corresponding functional groups are as follows. Peaks 3391 cm^−1^ and 1640 cm^−1^ are associated with the O–H deformation of water. Peak 1427 cm^−1^ agrees with the C–H bend. The wavenumber 980 cm^−1^ is associated with in-plane silicate stretching, while wavenumbers 726.8 cm^−1^ and 574 cm^−1^ correspond to the Si–O asymmetrical bending vibrations (Coates 2000; Jozanikohan and Abarghooei 2022). The minerals associated with particular wavenumbers and functional groups of the quarry dust are presented in Table 6. Based on the observed peaks, associated functional groups, and likely minerals, quarry dust is capable of replacing river sand in concrete making (Gnanasaravanan and Rajkumar 2013).

**Table 6:** Minerals and associated band groups in quarry dust

Minerals	Wave number (cm^−1^)	Band group
Kaolinite	3391	O–H deformation
Magnesium and chlorite	1640	O–H deformation
Carbonate	1457	C–H bend
Quartz	980	Si–O stretching in plane
Quartz and feldspar	726.8	Si–O asymmetrical bending
Quartz and feldspar	574	Si–O asymmetrical bending

(Coates 2000; Jozanikohan and Abarghooei 2022)

### Slump test results

Table 7 displays the results of the slump tests performed on the fresh concrete to find out the uniformity and workability of the blend.

**Table 7:** Effect of PSA and QD partial and full replacements on the workability of the concrete

Concrete designation	% partial replacements		Slump (mm)
	PSA	QD	
A	0	0	66.1
B	5	5	67.0
C	10	10	67.0
D	15	15	68.0
E	20	20	68.2
F	100	0	41.1
G	0	100	79.6
H	100	100	55.9

The effect of percentage partial and full replacements with PSA and QD on how workable the concrete can be is presented in Table 7. It could be observed that the increased % of replacements with PSA and QD yielded an increase in workability. This may be because it takes a longer time for the PSA cement to chemically react with water (hydration) than in the case of OPC cement and water, which could also cause the reduction in value as seen when 100% of PSA and highest slump obtained when 100% of cement was used with QD. The behavior agrees with that reported by Tayeh et al. (2020).

### Density of the concrete

**Fig. 5:**
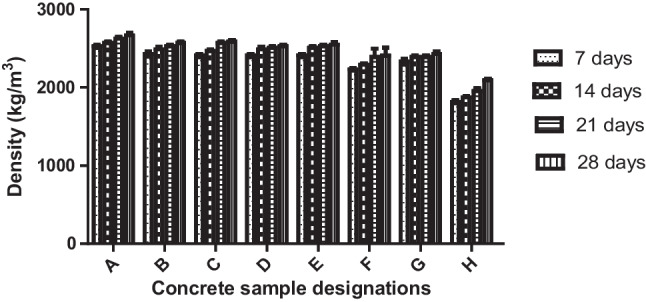
Variation of densities of the produced concrete samples

The test on the density of the concrete was used in measuring the unit weight of the concrete samples, and the result is revealed in Fig. 5. It could be understood that concrete A (control) without any replacement has a density slightly higher (3–5%) than the other concrete mixtures that were partially replaced with PSA and QD. This implies that partially replacing OPC and river sand with 5%, 10%, 15%, and 20% PSA and QD, respectively, slightly affects the concrete’s unit weight. The result agrees with that reported by Tayeh et al. (2020). Fully replacing OPC and river sand gave a remarkable reduction in the unit weights of the concrete samples, especially for days of curing that are not up to 28 days. Then as the days of curing increase, the unit weights for all the concrete samples increase, for more voids are filled with concrete mixes as the days of curing linger. According to the standard classification of concrete masonry units (ASTM 2017), concrete samples A–G, for all the days of curing, and sample H for 28 days of curing belong to the class of normal weight concrete, having densities that are more than 2000 kg/m^3^ (Eziefula et al. 2018). Then, concrete sample H, cured for 7, 14, and 21 days, is classified as medium-weight concrete with densities between 1680 and 2000 kg/m^3^.

### Compressive strength of concrete samples

**Fig. 6:**
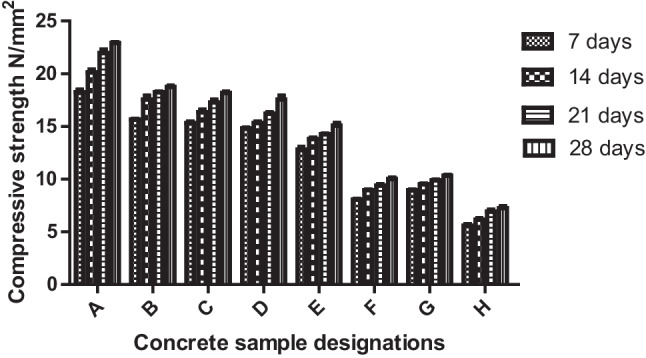
Variation of compressive strengths of the produced concrete

In Fig. 6, one can see the compressive strength of various concrete samples that were cured for different periods—7, 14, 21, and 28 days. The results indicate that the highest compressive strength was observed in all concrete samples after a curing period of 28 days. This suggests that the longer the concrete is cured, the more compression force it can carry. The concrete without waste addition showed the highest compressive strength; maybe there were more voids introduced in the bonds when the waste materials (PSA and QD) were added as against their non-inclusion (Kareem et al. 2022). Concerning the standard compressive strengths of concrete masonry units, concrete samples A–F, with partial replacements of PSA and QD, met the standard for having values greater than the minimum value of 13.8 MPa, for an average of 3 units. However, concrete samples G, H, and I, with full replacements, could not fit into the stipulated standard (ASTM 2017). Thus, concrete samples A–F for all the days of curing (7, 14, 21, and 28) are fit for applications that require both load-bearing and non-load-bearing activities (ASTM 2017).

### Connection between compressive strength and density and slump values

**Fig. 7:**
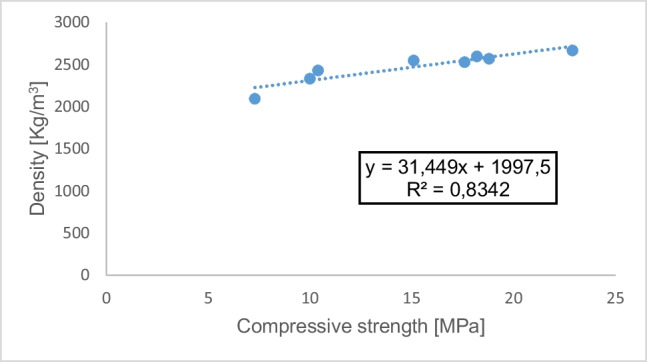
Relationship between compressive strength and density for the samples

**Fig. 8:**
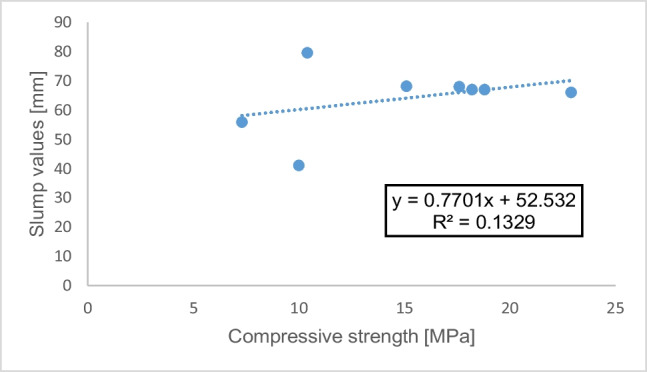
Relationship between compressive strength and slump values

The relationships between compressive strengths of the investigated samples of concrete cured in 28 days, the density, and slump values were investigated. The plots are shown in Figs. 7 and 8, respectively. It is worth noting that there exists a significant linear correlation between the density and compressive strength of a material with a correlation coefficient, *R* of 0.91 (since *R*^2^ = 0.8342), as shown in Fig. 7. It may not mean a cause-and-effect rapport between the variables but rather to show the degree of interconnection between them. The correlation coefficient, *R*, between compressive strength and slump values in Fig. 8 is 0.31 (since *R*^2^ = 0.1329), indicating a weak linear relationship. This demonstrates the degree of interconnection between the slump values and the compressive strength results (Bamigboye et al. 2020).

## Conclusion

This study examines the impact of replacing ordinary Portland cement (OPC) with periwinkle shell ash (PSA) and river sand with quarry dust (QD) at different weight percentages (5%, 10%, 15%, 20%, and 100%) on the physical and mechanical characteristics of concrete. The results from the study showed that with the increase in the partial replacements of OPC and sand, there was an improvement in how workable the concrete was. The samples without partial replacements showed a 3–5% increment in unit weights compared to those with partial replacements. The control sample gave the optimum compressive strength of 22.9 N/mm^2^, higher than those that were partially replaced, ranging between 18.8 and 15.1 N/mm^2^. It could be deduced that partial and full replacements with PSA and QD for Portland cement and river sand, respectively, can yield normal-weight concrete. Furthermore, partial replacements with PSA and QD produce concrete suitable for load and non-load-bearing applications. The usage of the waste materials would also lead to the reduction of the waste materials from polluting the environment, into useful concrete products.

## Data Availability

No datasets were generated or analyzed in this study.
